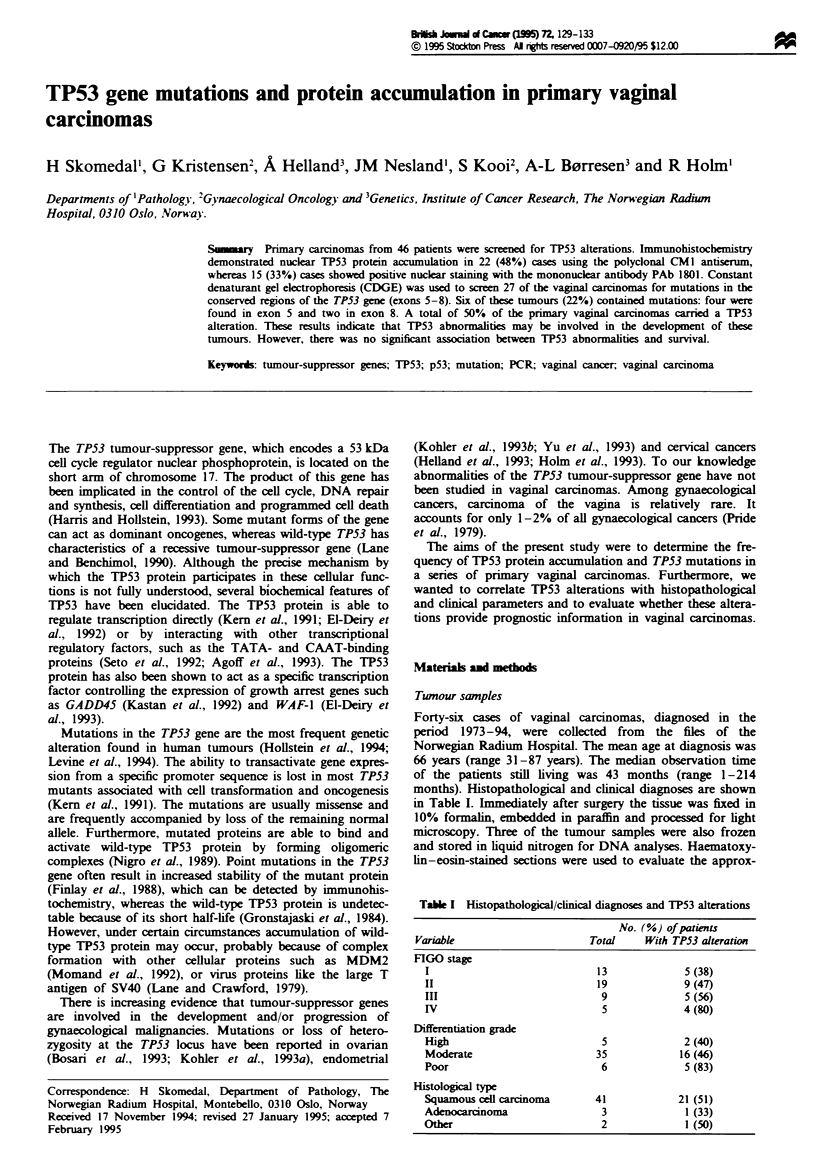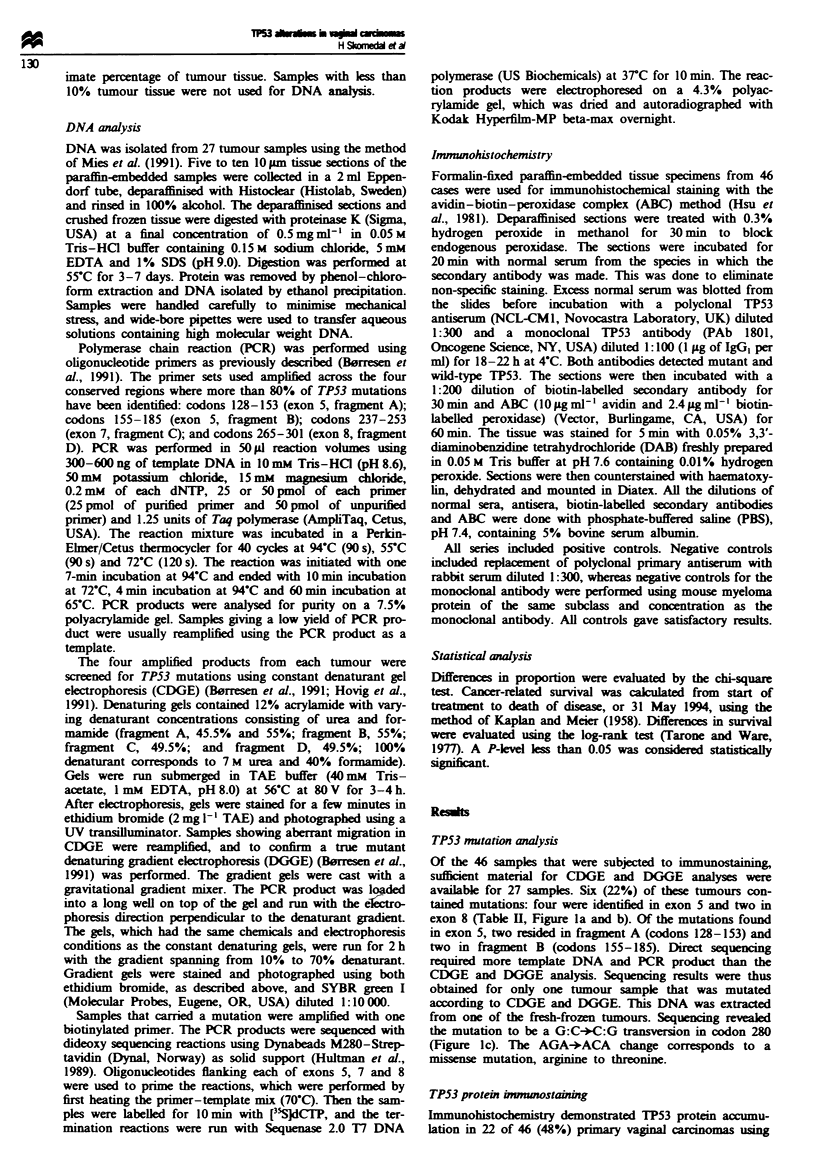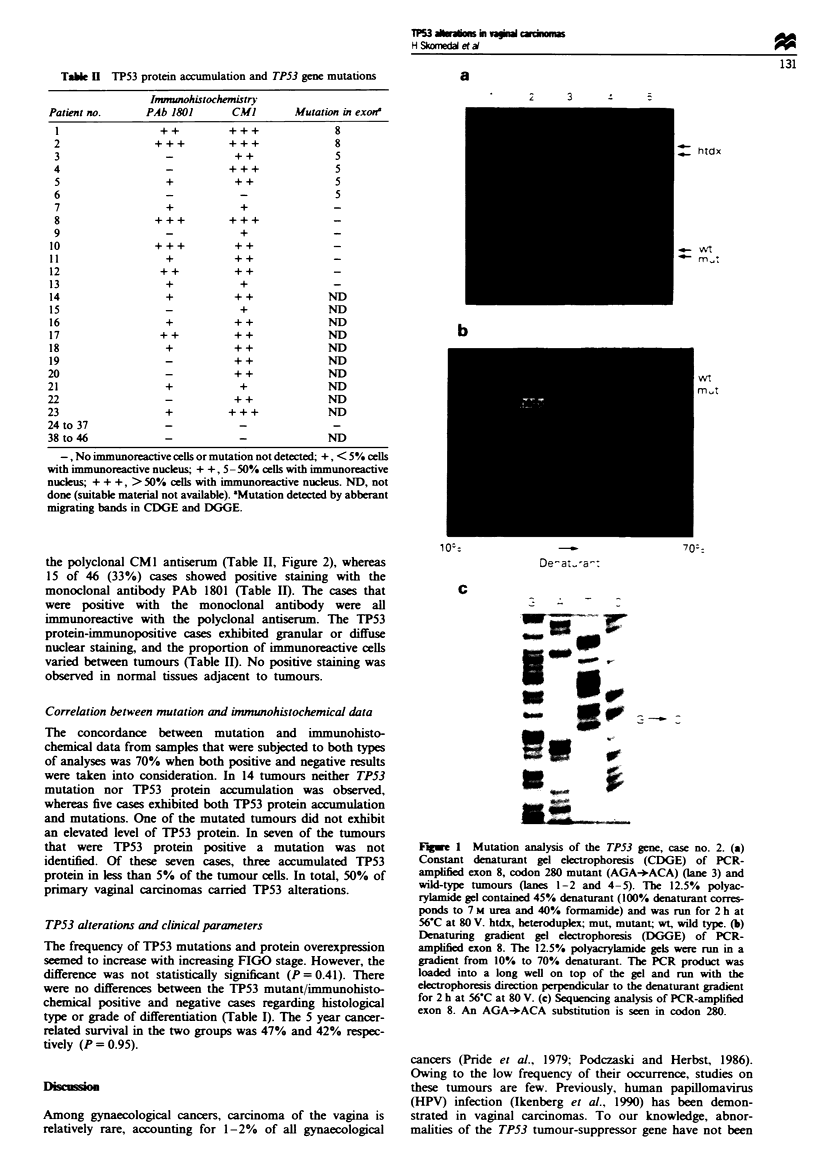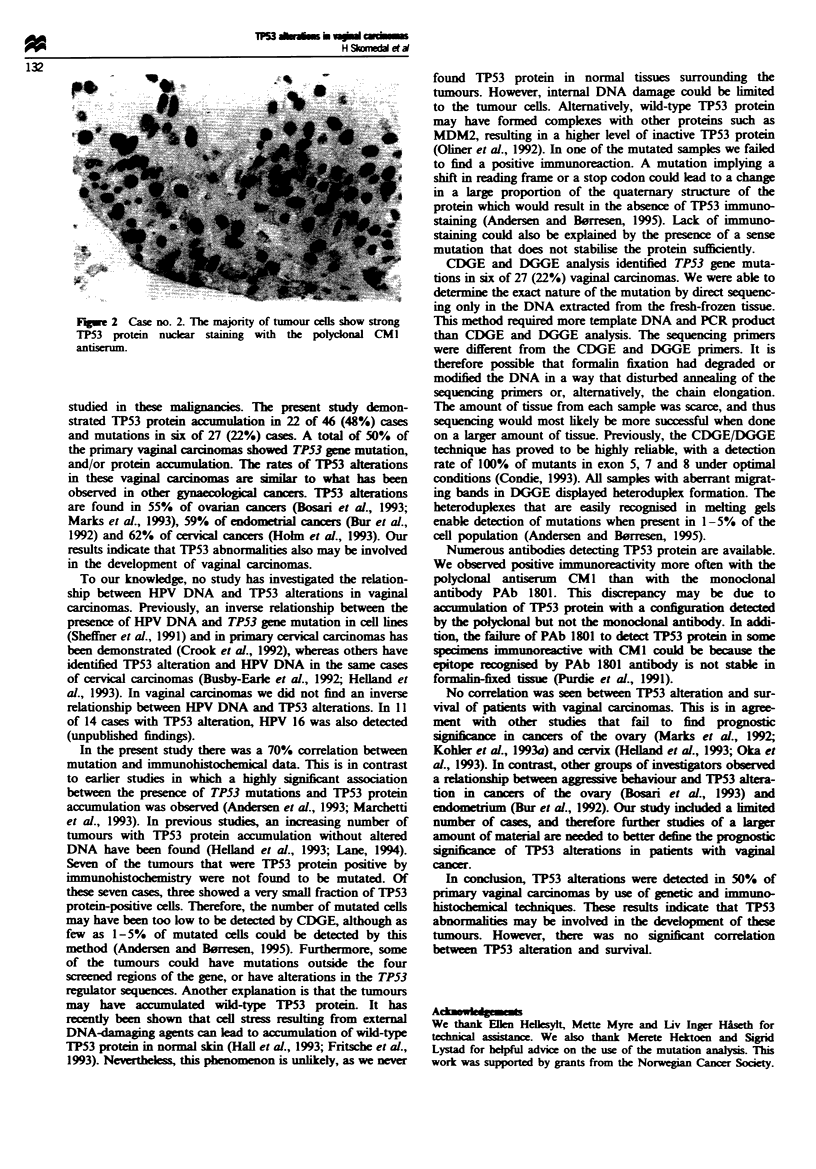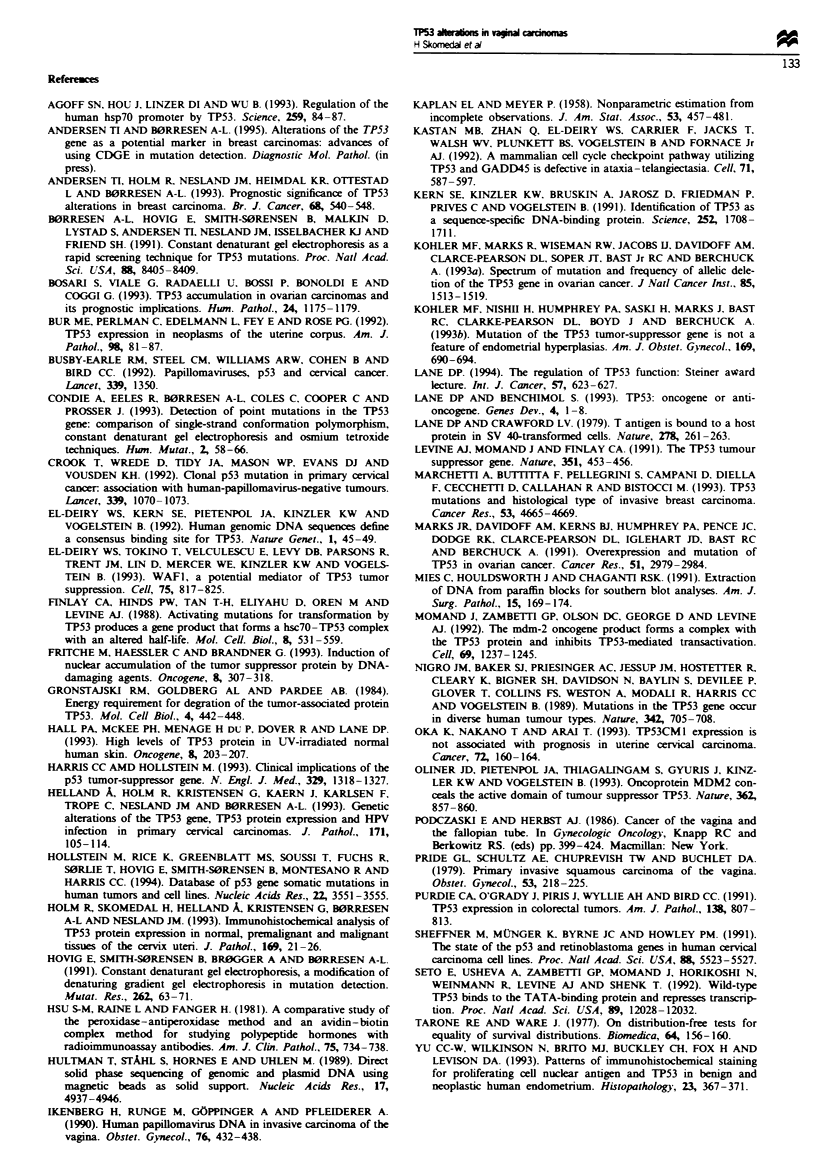# TP53 gene mutations and protein accumulation in primary vaginal carcinomas.

**DOI:** 10.1038/bjc.1995.288

**Published:** 1995-07

**Authors:** H. Skomedal, G. Kristensen, A. Helland, J. M. Nesland, S. Kooi, A. L. Børresen, R. Holm

**Affiliations:** Department of Pathology, Norwegian Radium Hospital, Oslo.

## Abstract

**Images:**


© 1995 Stockton Press All rights reserved 0007-0920/95 $12.00

### TP53 gene mutations and protein accumulation in primary vaginal carcinomas

H Skomedal[1], G Kristensen[2], Å Helland[3], JM Nesland[1], S Kooi[2], A-L Børresen[3] and R Holm[1]

*Departments of [1]Pathology, [2]Gynaecological Oncology and [3]Genetics, Institute of Cancer Research, The Norwegian Radium Hospital, 0310 Oslo, Norway.*

**Summary** Primary carcinomas from 46 patients were screened for TP53 alterations. Immunohistochemistry demonstrated nuclear TP53 protein accumulation in 22 (48%) cases using the polyclonal CM1 antiserum, whereas 15 (33%) cases showed positive nuclear staining with the mononuclear antibody PAb 1801. Constant denaturant gel electrophoresis (CDGE) was used to screen 27 of the vaginal carcinomas for mutations in the conserved regions of the *TP53* gene (exons 5-8). Six of these tumours (22%) contained mutations: four were found in exon 5 and two in exon 8. A total of 50% of the primary vaginal carcinomas carried a TP53 alteration. These results indicate that TP53 abnormalities may be involved in the development of these tumours. However, there was no significant association between TP53 abnormalities and survival.

**Keywords**: tumour-suppressor genes; TP53; p53; mutation; PCR; vaginal cancer; vaginal carcinoma

The *TP53* tumour-suppressor gene, which encodes a 53 kDa cell cycle regulator nuclear phosphoprotein, is located on the short arm of chromosome 17. The product of this gene has been implicated in the control of the cell cycle, DNA repair and synthesis, cell differentiation and programmed cell death (Harris and Hollstein, 1993). Some mutant forms of the gene can act as dominant oncogenes, whereas wild-type *TP53* has characteristics of a recessive tumour-suppressor gene (Lane and Benchimol, 1990). Although the precise mechanism by which the TP53 protein participates in these cellular functions is not fully understood, several biochemical features of TP53 have been elucidated. The TP53 protein is able to regulate transcription directly (Kern *et al.*, 1991; El-Deiry *et al.*, 1992) or by interacting with other transcriptional regulatory factors, such as the TATA- and CAAT-binding proteins (Seto *et al.*, 1992; Agoff *et al.*, 1993). The TP53 protein has also been shown to act as a specific transcription factor controlling the expression of growth arrest genes such as *GADD45* (Kastan *et al.*, 1992) and *WAF*-1 (El-Deiry *et al.*, 1993).

Mutations in the *TP53* gene are the most frequent genetic alteration found in human tumours (Hollstein *et al.*, 1994; Levine *et al.*, 1994). The ability to transactivate gene expression from a specific promoter sequence is lost in most *TP53* mutants associated with cell transformation and oncogenesis (Kern *et al.*, 1991). The mutations are usually missense and are frequently accompanied by loss of the remaining normal allele. Furthermore, mutated proteins are able to bind and activate wild-type TP53 protein by forming oligomeric complexes (Nigro *et al.*, 1989). Point mutations in the *TP53* gene often result in increased stability of the mutant protein (Finlay *et al.*, 1988), which can be detected by immunohistochemistry, whereas the wild-type TP53 protein is undetectable because of its short half-life (Gronstajaski *et al.*, 1984). However, under certain circumstances accumulation of wild-type TP53 protein may occur, probably because of complex formation with other cellular proteins such as MDM2 (Momand *et al.*, 1992), or virus proteins like the large T antigen of SV40 (Lane and Crawford, 1979).

There is increasing evidence that tumour-suppressor genes are involved in the development and/or progression of gynaecological malignancies. Mutations or loss of heterozygosity at the *TP53* locus have been reported in ovarian (Bosari *et al.*, 1993; Kohler *et al.*, 1993*a*), endometrial (Kohler *et al.*, 1993*b*; Yu *et al.*, 1993) and cervical cancers (Helland *et al.*, 1993; Holm *et al.*, 1993). To our knowledge abnormalities of the *TP53* tumour-suppressor gene have not been studied in vaginal carcinomas. Among gynaecological cancers, carcinoma of the vagina is relatively rare. It accounts for only 1-2% of all gynaecological cancers (Pride *et al.*, 1979).

The aims of the present study were to determine the frequency of TP53 protein accumulation and *TP53* mutations in a series of primary vaginal carcinomas. Furthermore, we wanted to correlate TP53 alterations with histopathological and clinical parameters and to evaluate whether these alterations provide prognostic information in vaginal carcinomas.

### Materials and methods

### *Tumour samples*

Forty-six cases of vaginal carcinomas, diagnosed in the period 1973-94, were collected from the files of the Norwegian Radium Hospital. The mean age at diagnosis was 66 years (range 31-87 years). The median observation time of the patients still living was 43 months (range 1-214 months). Histopathological and clinical diagnoses are shown in Table I. Immediately after surgery the tissue was fixed in 10% formalin, embedded in paraffin and processed for light microscopy. Three of the tumour samples were also frozen and stored in liquid nitrogen for DNA analyses. Haematoxylin-eosin-stained sections were used to evaluate the approx-

**Table I** Histopathological/clinical diagnoses and TP53 alterations

| *Variable* | *No. (%) of patients* *Total* | *With TP53 alteration* |
|---|---|---|
| FIGO stage | | |
| I | 13 | 5 (38) |
| II | 19 | 9 (47) |
| III | 9 | 5 (56) |
| IV | 5 | 4 (80) |
| Differentiation grade | | |
| High | 5 | 2 (40) |
| Moderate | 35 | 16 (46) |
| Poor | 6 | 5 (83) |
| Histological type | | |
| Squamous cell carcinoma | 41 | 21 (51) |
| Adenocarcinoma | 3 | 1 (33) |
| Other | 2 | 1 (50) |

Correspondence: H Skomedal, Department of Pathology, The Norwegian Radium Hospital, Montebello, 0310 Oslo, Norway
Received 17 November 1994; revised 27 January 1995; accepted 7 February 1995

imate percentage of tumour tissue. Samples with less than 10% tumour tissue were not used for DNA analysis.

*DNA analysis*

DNA was isolated from 27 tumour samples using the method of Mies *et al.* (1991). Five to ten 10 μm tissue sections of the paraffin-embedded samples were collected in a 2 ml Eppendorf tube, deparaffinised with Histoclear (Histolab, Sweden) and rinsed in 100% alcohol. The deparaffinised sections and crushed frozen tissue were digested with proteinase K (Sigma, USA) at a final concentration of 0.5 mg $ml^{-1}$ in 0.05 M Tris-HCl buffer containing 0.15 M sodium chloride, 5 mM EDTA and 1% SDS (pH 9.0). Digestion was performed at 55°C for 3-7 days. Protein was removed by phenol-chloroform extraction and DNA isolated by ethanol precipitation. Samples were handled carefully to minimise mechanical stress, and wide-bore pipettes were used to transfer aqueous solutions containing high molecular weight DNA.

Polymerase chain reaction (PCR) was performed using oligonucleotide primers as previously described (Børresen *et al.*, 1991). The primer sets used amplified across the four conserved regions where more than 80% of *TP53* mutations have been identified: codons 128-153 (exon 5, fragment A); codons 155-185 (exon 5, fragment B); codons 237-253 (exon 7, fragment C); and codons 265-301 (exon 8, fragment D). PCR was performed in 50 μl reaction volumes using 300-600 ng of template DNA in 10 mM Tris-HCl (pH 8.6), 50 mM potassium chloride, 15 mM magnesium chloride, 0.2 mM of each dNTP, 25 or 50 pmol of each primer (25 pmol of purified primer and 50 pmol of unpurified primer) and 1.25 units of *Taq* polymerase (AmpliTaq, Cetus, USA). The reaction mixture was incubated in a Perkin-Elmer/Cetus thermocycler for 40 cycles at 94°C (90 s), 55°C (90 s) and 72°C (120 s). The reaction was initiated with one 7-min incubation at 94°C and ended with 10 min incubation at 72°C, 4 min incubation at 94°C and 60 min incubation at 65°C. PCR products were analysed for purity on a 7.5% polyacrylamide gel. Samples giving a low yield of PCR product were usually reamplified using the PCR product as a template.

The four amplified products from each tumour were screened for *TP53* mutations using constant denaturant gel electrophoresis (CDGE) (Børresen *et al.*, 1991; Hovig *et al.*, 1991). Denaturing gels contained 12% acrylamide with varying denaturant concentrations consisting of urea and formamide (fragment A, 45.5% and 55%; fragment B, 55%; fragment C, 49.5%; and fragment D, 49.5%; 100% denaturant corresponds to 7 M urea and 40% formamide). Gels were run submerged in TAE buffer (40 mM Tris-acetate, 1 mM EDTA, pH 8.0) at 56°C at 80 V for 3-4 h. After electrophoresis, gels were stained for a few minutes in ethidium bromide (2 mg $l^{-1}$ TAE) and photographed using a UV transilluminator. Samples showing aberrant migration in CDGE were reamplified, and to confirm a true mutant denaturing gradient electrophoresis (DGGE) (Børresen *et al.*, 1991) was performed. The gradient gels were cast with a gravitational gradient mixer. The PCR product was loaded into a long well on top of the gel and run with the electrophoresis direction perpendicular to the denaturant gradient. The gels, which had the same chemicals and electrophoresis conditions as the constant denaturing gels, were run for 2 h with the gradient spanning from 10% to 70% denaturant. Gradient gels were stained and photographed using both ethidium bromide, as described above, and SYBR green I (Molecular Probes, Eugene, OR, USA) diluted 1:10 000.

Samples that carried a mutation were amplified with one biotinylated primer. The PCR products were sequenced with dideoxy sequencing reactions using Dynabeads M280-Streptavidin (Dynal, Norway) as solid support (Hultman *et al.*, 1989). Oligonucleotides flanking each of exons 5, 7 and 8 were used to prime the reactions, which were performed by first heating the primer-template mix (70°C). Then the samples were labelled for 10 min with [$^{35}$S]dCTP, and the termination reactions were run with Sequenase 2.0 T7 DNA polymerase (US Biochemicals) at 37°C for 10 min. The reaction products were electrophoresed on a 4.3% polyacrylamide gel, which was dried and autoradiographed with Kodak Hyperfilm-MP beta-max overnight.

*Immunohistochemistry*

Formalin-fixed paraffin-embedded tissue specimens from 46 cases were used for immunohistochemical staining with the avidin-biotin-peroxidase complex (ABC) method (Hsu *et al.*, 1981). Deparaffinised sections were treated with 0.3% hydrogen peroxide in methanol for 30 min to block endogenous peroxidase. The sections were incubated for 20 min with normal serum from the species in which the secondary antibody was made. This was done to eliminate non-specific staining. Excess normal serum was blotted from the slides before incubation with a polyclonal TP53 antiserum (NCL-CM1, Novocastra Laboratory, UK) diluted 1:300 and a monoclonal TP53 antibody (PAb 1801, Oncogene Science, NY, USA) diluted 1:100 (1 μg of $IgG_1$ per ml) for 18-22 h at 4°C. Both antibodies detected mutant and wild-type TP53. The sections were then incubated with a 1:200 dilution of biotin-labelled secondary antibody for 30 min and ABC (10 μg $ml^{-1}$ avidin and 2.4 μg $ml^{-1}$ biotin-labelled peroxidase) (Vector, Burlingame, CA, USA) for 60 min. The tissue was stained for 5 min with 0.05% 3,3'-diaminobenzidine tetrahydrochloride (DAB) freshly prepared in 0.05 M Tris buffer at pH 7.6 containing 0.01% hydrogen peroxide. Sections were then counterstained with haematoxylin, dehydrated and mounted in Diatex. All the dilutions of normal sera, antisera, biotin-labelled secondary antibodies and ABC were done with phosphate-buffered saline (PBS), pH 7.4, containing 5% bovine serum albumin.

All series included positive controls. Negative controls included replacement of polyclonal primary antiserum with rabbit serum diluted 1:300, whereas negative controls for the monoclonal antibody were performed using mouse myeloma protein of the same subclass and concentration as the monoclonal antibody. All controls gave satisfactory results.

*Statistical analysis*

Differences in proportion were evaluated by the chi-square test. Cancer-related survival was calculated from start of treatment to death of disease, or 31 May 1994, using the method of Kaplan and Meier (1958). Differences in survival were evaluated using the log-rank test (Tarone and Ware, 1977). A *P*-level less than 0.05 was considered statistically significant.

### Results

*TP53 mutation analysis*

Of the 46 samples that were subjected to immunostaining, sufficient material for CDGE and DGGE analyses were available for 27 samples. Six (22%) of these tumours contained mutations: four were identified in exon 5 and two in exon 8 (Table II, Figure 1a and b). Of the mutations found in exon 5, two resided in fragment A (codons 128-153) and two in fragment B (codons 155-185). Direct sequencing required more template DNA and PCR product than the CDGE and DGGE analysis. Sequencing results were thus obtained for only one tumour sample that was mutated according to CDGE and DGGE. This DNA was extracted from one of the fresh-frozen tumours. Sequencing revealed the mutation to be a G:C→C:G transversion in codon 280 (Figure 1c). The AGA→ACA change corresponds to a missense mutation, arginine to threonine.

*TP53 protein immunostaining*

Immunohistochemistry demonstrated TP53 protein accumulation in 22 of 46 (48%) primary vaginal carcinomas using

**Table II** TP53 protein accumulation and *TP53* gene mutations

| | *Immunohistochemistry* | | |
|---|---|---|---|
| *Patient no.* | *PAb 1801* | *CM1* | *Mutation in exon*[a] |
| 1 | + + | + + + | 8 |
| 2 | + + + | + + + | 8 |
| 3 | – | + + | 5 |
| 4 | – | + + + | 5 |
| 5 | + | + + | 5 |
| 6 | – | – | 5 |
| 7 | + | + | – |
| 8 | + + + | + + + | – |
| 9 | – | + | – |
| 10 | + + + | + + | – |
| 11 | + | + + | – |
| 12 | + + | + + | – |
| 13 | + | + | – |
| 14 | + | + + | ND |
| 15 | – | + | ND |
| 16 | + | + + | ND |
| 17 | + + | + + | ND |
| 18 | + | + + | ND |
| 19 | – | + + | ND |
| 20 | – | + + | ND |
| 21 | + | + | ND |
| 22 | – | + + | ND |
| 23 | + | + + + | ND |
| 24 to 37 | – | – | – |
| 38 to 46 | – | – | ND |

–, No immunoreactive cells or mutation not detected; +, <5% cells with immunoreactive nucleus; + +, 5–50% cells with immunoreactive nucleus; + + +, >50% cells with immunoreactive nucleus. ND, not done (suitable material not available). [a]Mutation detected by abberant migrating bands in CDGE and DGGE.

the polyclonal CM1 antiserum (Table II, Figure 2), whereas 15 of 46 (33%) cases showed positive staining with the monoclonal antibody PAb 1801 (Table II). The cases that were positive with the monoclonal antibody were all immunoreactive with the polyclonal antiserum. The TP53 protein-immunopositive cases exhibited granular or diffuse nuclear staining, and the proportion of immunoreactive cells varied between tumours (Table II). No positive staining was observed in normal tissues adjacent to tumours.

### *Correlation between mutation and immunohistochemical data*

The concordance between mutation and immunohistochemical data from samples that were subjected to both types of analyses was 70% when both positive and negative results were taken into consideration. In 14 tumours neither *TP53* mutation nor TP53 protein accumulation was observed, whereas five cases exhibited both TP53 protein accumulation and mutations. One of the mutated tumours did not exhibit an elevated level of TP53 protein. In seven of the tumours that were TP53 protein positive a mutation was not identified. Of these seven cases, three accumulated TP53 protein in less than 5% of the tumour cells. In total, 50% of primary vaginal carcinomas carried TP53 alterations.

### *TP53 alterations and clinical parameters*

The frequency of TP53 mutations and protein overexpression seemed to increase with increasing FIGO stage. However, the difference was not statistically significant ($P = 0.41$). There were no differences between the TP53 mutant/immunohistochemical positive and negative cases regarding histological type or grade of differentiation (Table I). The 5 year cancer-related survival in the two groups was 47% and 42% respectively ($P = 0.95$).

### Discussion

Among gynaecological cancers, carcinoma of the vagina is relatively rare, accounting for 1–2% of all gynaecological

**Figure 1** Mutation analysis of the *TP53* gene, case no. 2. (**a**) Constant denaturant gel electrophoresis (CDGE) of PCR-amplified exon 8, codon 280 mutant (AGA→ACA) (lane 3) and wild-type tumours (lanes 1–2 and 4–5). The 12.5% polyacrylamide gel contained 45% denaturant (100% denaturant corresponds to 7 M urea and 40% formamide) and was run for 2 h at 56°C at 80 V. htdx, heteroduplex; mut, mutant; wt, wild type. (**b**) Denaturing gradient gel electrophoresis (DGGE) of PCR-amplified exon 8. The 12.5% polyacrylamide gels were run in a gradient from 10% to 70% denaturant. The PCR product was loaded into a long well on top of the gel and run with the electrophoresis direction perpendicular to the denaturant gradient for 2 h at 56°C at 80 V. (**c**) Sequencing analysis of PCR-amplified exon 8. An AGA→ACA substitution is seen in codon 280.

cancers (Pride *et al*., 1979; Podczaski and Herbst, 1986). Owing to the low frequency of their occurrence, studies on these tumours are few. Previously, human papillomavirus (HPV) infection (Ikenberg *et al*., 1990) has been demonstrated in vaginal carcinomas. To our knowledge, abnormalities of the *TP53* tumour-suppressor gene have not been

Figure 2 Case no. 2. The majority of tumour cells show strong TP53 protein nuclear staining with the polyclonal CM1 antiserum.

studied in these malignancies. The present study demonstrated TP53 protein accumulation in 22 of 46 (48%) cases and mutations in six of 27 (22%) cases. A total of 50% of the primary vaginal carcinomas showed *TP53* gene mutation, and/or protein accumulation. The rates of TP53 alterations in these vaginal carcinomas are similar to what has been observed in other gynaecological cancers. TP53 alterations are found in 55% of ovarian cancers (Bosari *et al*., 1993; Marks *et al*., 1993), 59% of endometrial cancers (Bur *et al*., 1992) and 62% of cervical cancers (Holm *et al*., 1993). Our results indicate that TP53 abnormalities also may be involved in the development of vaginal carcinomas.

To our knowledge, no study has investigated the relationship between HPV DNA and TP53 alterations in vaginal carcinomas. Previously, an inverse relationship between the presence of HPV DNA and *TP53* gene mutation in cell lines (Sheffner *et al*., 1991) and in primary cervical carcinomas has been demonstrated (Crook *et al*., 1992), whereas others have identified TP53 alteration and HPV DNA in the same cases of cervical carcinomas (Busby-Earle *et al*., 1992; Helland *et al*., 1993). In vaginal carcinomas we did not find an inverse relationship between HPV DNA and TP53 alterations. In 11 of 14 cases with TP53 alteration, HPV 16 was also detected (unpublished findings).

In the present study there was a 70% correlation between mutation and immunohistochemical data. This is in contrast to earlier studies in which a highly significant association between the presence of *TP53* mutations and TP53 protein accumulation was observed (Andersen *et al*., 1993; Marchetti *et al*., 1993). In previous studies, an increasing number of tumours with TP53 protein accumulation without altered DNA have been found (Helland *et al*., 1993; Lane, 1994). Seven of the tumours that were TP53 protein positive by immunohistochemistry were not found to be mutated. Of these seven cases, three showed a very small fraction of TP53 protein-positive cells. Therefore, the number of mutated cells may have been too low to be detected by CDGE, although as few as 1–5% of mutated cells could be detected by this method (Andersen and Børresen, 1995). Furthermore, some of the tumours could have mutations outside the four screened regions of the gene, or have alterations in the *TP53* regulator sequences. Another explanation is that the tumours may have accumulated wild-type TP53 protein. It has recently been shown that cell stress resulting from external DNA-damaging agents can lead to accumulation of wild-type TP53 protein in normal skin (Hall *et al*., 1993; Fritsche *et al*., 1993). Nevertheless, this phenomenon is unlikely, as we never found TP53 protein in normal tissues surrounding the tumours. However, internal DNA damage could be limited to the tumour cells. Alternatively, wild-type TP53 protein may have formed complexes with other proteins such as MDM2, resulting in a higher level of inactive TP53 protein (Oliner *et al*., 1992). In one of the mutated samples we failed to find a positive immunoreaction. A mutation implying a shift in reading frame or a stop codon could lead to a change in a large proportion of the quaternary structure of the protein which would result in the absence of TP53 immunostaining (Andersen and Børresen, 1995). Lack of immunostaining could also be explained by the presence of a sense mutation that does not stabilise the protein sufficiently.

CDGE and DGGE analysis identified *TP53* gene mutations in six of 27 (22%) vaginal carcinomas. We were able to determine the exact nature of the mutation by direct sequencing only in the DNA extracted from the fresh-frozen tissue. This method required more template DNA and PCR product than CDGE and DGGE analysis. The sequencing primers were different from the CDGE and DGGE primers. It is therefore possible that formalin fixation had degraded or modified the DNA in a way that disturbed annealing of the sequencing primers or, alternatively, the chain elongation. The amount of tissue from each sample was scarce, and thus sequencing would most likely be more successful when done on a larger amount of tissue. Previously, the CDGE/DGGE technique has proved to be highly reliable, with a detection rate of 100% of mutants in exon 5, 7 and 8 under optimal conditions (Condie, 1993). All samples with aberrant migrating bands in DGGE displayed heteroduplex formation. The heteroduplexes that are easily recognised in melting gels enable detection of mutations when present in 1–5% of the cell population (Andersen and Børresen, 1995).

Numerous antibodies detecting TP53 protein are available. We observed positive immunoreactivity more often with the polyclonal antiserum CM1 than with the monoclonal antibody PAb 1801. This discrepancy may be due to accumulation of TP53 protein with a configuration detected by the polyclonal but not the monoclonal antibody. In addition, the failure of PAb 1801 to detect TP53 protein in some specimens immunoreactive with CM1 could be because the epitope recognised by PAb 1801 antibody is not stable in formalin-fixed tissue (Purdie *et al*., 1991).

No correlation was seen between TP53 alteration and survival of patients with vaginal carcinomas. This is in agreement with other studies that fail to find prognostic significance in cancers of the ovary (Marks *et al*., 1992; Kohler *et al*., 1993*a*) and cervix (Helland *et al*., 1993; Oka *et al*., 1993). In contrast, other groups of investigators observed a relationship between aggressive behaviour and TP53 alteration in cancers of the ovary (Bosari *et al*., 1993) and endometrium (Bur *et al*., 1992). Our study included a limited number of cases, and therefore further studies of a larger amount of material are needed to better define the prognostic significance of TP53 alterations in patients with vaginal cancer.

In conclusion, TP53 alterations were detected in 50% of primary vaginal carcinomas by use of genetic and immunohistochemical techniques. These results indicate that TP53 abnormalities may be involved in the development of these tumours. However, there was no significant correlation between TP53 alteration and survival.

**Acknowledgements**

We thank Ellen Hellesylt, Mette Myre and Liv Inger Håseth for technical assistance. We also thank Merete Hektoen and Sigrid Lystad for helpful advice on the use of the mutation analysis. This work was supported by grants from the Norwegian Cancer Society.

**References**

AGOFF SN, HOU J, LINZER DI AND WU B. (1993). Regulation of the human hsp70 promoter by TP53. *Science*, **259**, 84-87.

ANDERSEN TI AND BØRRESEN A-L. (1995). Alterations of the *TP53* gene as a potential marker in breast carcinomas: advances of using CDGE in mutation detection. *Diagnostic Mol. Pathol.* (in press).

ANDERSEN TI, HOLM R, NESLAND JM, HEIMDAL KR, OTTESTAD L AND BØRRESEN A-L. (1993). Prognostic significance of TP53 alterations in breast carcinoma. *Br. J. Cancer*, **68**, 540-548.

BØRRESEN A-L, HOVIG E, SMITH-SØRENSEN B, MALKIN D, LYSTAD S, ANDERSEN TI, NESLAND JM, ISSELBACHER KJ AND FRIEND SH. (1991). Constant denaturant gel electrophoresis as a rapid screening technique for TP53 mutations. *Proc. Natl Acad. Sci. USA*, **88**, 8405-8409.

BOSARI S, VIALE G, RADAELLI U, BOSSI P, BONOLDI E AND COGGI G. (1993). TP53 accumulation in ovarian carcinomas and its prognostic implications. *Hum. Pathol.*, **24**, 1175-1179.

BUR ME, PERLMAN C, EDELMANN L, FEY E AND ROSE PG. (1992). TP53 expression in neoplasms of the uterine corpus. *Am. J. Pathol.*, **98**, 81-87.

BUSBY-EARLE RM, STEEL CM, WILLIAMS ARW, COHEN B AND BIRD CC. (1992). Papillomaviruses, p53 and cervical cancer. *Lancet*, **339**, 1350.

CONDIE A, EELES R, BØRRESEN A-L, COLES C, COOPER C AND PROSSER J. (1993). Detection of point mutations in the TP53 gene: comparison of single-strand conformation polymorphism, constant denaturant gel electrophoresis and osmium tetroxide techniques. *Hum. Mutat.*, **2**, 58-66.

CROOK T, WREDE D, TIDY JA, MASON WP, EVANS DJ AND VOUSDEN KH. (1992). Clonal p53 mutation in primary cervical cancer: association with human-papillomavirus-negative tumours. *Lancet*, **339**, 1070-1073.

EL-DEIRY WS, KERN SE, PIETENPOL JA, KINZLER KW AND VOGELSTEIN B. (1992). Human genomic DNA sequences define a consensus binding site for TP53. *Nature Genet.*, **1**, 45-49.

EL-DEIRY WS, TOKINO T, VELCULESCU E, LEVY DB, PARSONS R, TRENT JM, LIN D, MERCER WE, KINZLER KW AND VOGELSTEIN B. (1993). WAF1, a potential mediator of TP53 tumor suppression. *Cell*, **75**, 817-825.

FINLAY CA, HINDS PW, TAN T-H, ELIYAHU D, OREN M AND LEVINE AJ. (1988). Activating mutations for transformation by TP53 produces a gene product that forms a hsc70-TP53 complex with an altered half-life. *Mol. Cell. Biol.*, **8**, 531-559.

FRITCHE M, HAESSLER C AND BRANDNER G. (1993). Induction of nuclear accumulation of the tumor suppressor protein by DNA-damaging agents. *Oncogene*, **8**, 307-318.

GRONSTAJSKI RM, GOLDBERG AL AND PARDEE AB. (1984). Energy requirement for degration of the tumor-associated protein TP53. *Mol. Cell Biol.*, **4**, 442-448.

HALL PA, MCKEE PH, MENAGE H DU P, DOVER R AND LANE DP. (1993). High levels of TP53 protein in UV-irradiated normal human skin. *Oncogene*, **8**, 203-207.

HARRIS CC AMD HOLLSTEIN M. (1993). Clinical implications of the p53 tumor-suppressor gene. *N. Engl. J. Med.*, **329**, 1318-1327.

HELLAND Å, HOLM R, KRISTENSEN G, KAERN J, KARLSEN F, TROPE C, NESLAND JM AND BØRRESEN A-L. (1993). Genetic alterations of the TP53 gene, TP53 protein expression and HPV infection in primary cervical carcinomas. *J. Pathol.*, **171**, 105-114.

HOLLSTEIN M, RICE K, GREENBLATT MS, SOUSSI T, FUCHS R, SØRLIE T, HOVIG E, SMITH-SØRENSEN B, MONTESANO R AND HARRIS CC. (1994). Database of p53 gene somatic mutations in human tumors and cell lines. *Nucleic Acids Res.*, **22**, 3551-3555.

HOLM R, SKOMEDAL H, HELLAND Å, KRISTENSEN G, BØRRESEN A-L AND NESLAND JM. (1993). Immunohistochemical analysis of TP53 protein expression in normal, premalignant and malignant tissues of the cervix uteri. *J. Pathol.*, **169**, 21-26.

HOVIG E, SMITH-SØRENSEN B, BRØGGER A AND BØRRESEN A-L. (1991). Constant denaturant gel electrophoresis, a modification of denaturing gradient gel electrophoresis in mutation detection. *Mutat. Res.*, **262**, 63-71.

HSU S-M, RAINE L AND FANGER H. (1981). A comparative study of the peroxidase-antiperoxidase method and an avidin-biotin complex method for studying polypeptide hormones with radioimmunoassay antibodies. *Am. J. Clin. Pathol.*, **75**, 734-738.

HULTMAN T, STÅHL S, HORNES E AND UHLEN M. (1989). Direct solid phase sequencing of genomic and plasmid DNA using magnetic beads as solid support. *Nucleic Acids Res.*, **17**, 4937-4946.

IKENBERG H, RUNGE M, GÖPPINGER A AND PFLEIDERER A. (1990). Human papillomavirus DNA in invasive carcinoma of the vagina. *Obstet. Gynecol.*, **76**, 432-438.

KAPLAN EL AND MEYER P. (1958). Nonparametric estimation from incomplete observations. *J. Am. Stat. Assoc.*, **53**, 457-481.

KASTAN MB, ZHAN Q, EL-DEIRY WS, CARRIER F, JACKS T, WALSH WV, PLUNKETT BS, VOGELSTEIN B AND FORNACE Jr AJ. (1992). A mammalian cell cycle checkpoint pathway utilizing TP53 and GADD45 is defective in ataxia-telangiectasia. *Cell*, **71**, 587-597.

KERN SE, KINZLER KW, BRUSKIN A, JAROSZ D, FRIEDMAN P, PRIVES C AND VOGELSTEIN B. (1991). Identification of TP53 as a sequence-specific DNA-binding protein. *Science*, **252**, 1708-1711.

KOHLER MF, MARKS R, WISEMAN RW, JACOBS IJ, DAVIDOFF AM, CLARCE-PEARSON DL, SOPER JT, BAST Jr RC AND BERCHUCK A. (1993*a*). Spectrum of mutation and frequency of allelic deletion of the TP53 gene in ovarian cancer. *J Natl Cancer Inst.*, **85**, 1513-1519.

KOHLER MF, NISHII H, HUMPHREY PA, SASKI H, MARKS J, BAST RC, CLARKE-PEARSON DL, BOYD J AND BERCHUCK A. (1993*b*). Mutation of the TP53 tumor-suppressor gene is not a feature of endometrial hyperplasias. *Am. J. Obstet. Gynecol.*, **169**, 690-694.

LANE DP. (1994). The regulation of TP53 function: Steiner award lecture. *Int. J. Cancer*, **57**, 623-627.

LANE DP AND BENCHIMOL S. (1993). TP53: oncogene or anti-oncogene. *Genes Dev.*, **4**, 1-8.

LANE DP AND CRAWFORD LV. (1979). T antigen is bound to a host protein in SV 40-transformed cells. *Nature*, **278**, 261-263.

LEVINE AJ, MOMAND J AND FINLAY CA. (1991). The TP53 tumour suppressor gene. *Nature*, **351**, 453-456.

MARCHETTI A, BUTTITTA F, PELLEGRINI S, CAMPANI D, DIELLA F, CECCHETTI D, CALLAHAN R AND BISTOCCI M. (1993). TP53 mutations and histological type of invasive breast carcinoma. *Cancer Res.*, **53**, 4665-4669.

MARKS JR, DAVIDOFF AM, KERNS BJ, HUMPHREY PA, PENCE JC, DODGE RK, CLARCE-PEARSON DL, IGLEHART JD, BAST RC AND BERCHUCK A. (1991). Overexpression and mutation of TP53 in ovarian cancer. *Cancer Res.*, **51**, 2979-2984.

MIES C, HOULDSWORTH J AND CHAGANTI RSK. (1991). Extraction of DNA from paraffin blocks for southern blot analyses. *Am. J. Surg. Pathol.*, **15**, 169-174.

MOMAND J, ZAMBETTI GP, OLSON DC, GEORGE D AND LEVINE AJ. (1992). The mdm-2 oncogene product forms a complex with the TP53 protein and inhibits TP53-mediated transactivation. *Cell*, **69**, 1237-1245.

NIGRO JM, BAKER SJ, PRIESINGER AC, JESSUP JM, HOSTETTER R, CLEARY K, BIGNER SH, DAVIDSON N, BAYLIN S, DEVILEE P, GLOVER T, COLLINS FS, WESTON A, MODALI R, HARRIS CC AND VOGELSTEIN B. (1989). Mutations in the TP53 gene occur in diverse human tumour types. *Nature*, **342**, 705-708.

OKA K, NAKANO T AND ARAI T. (1993). TP53CM1 expression is not associated with prognosis in uterine cervical carcinoma. *Cancer*, **72**, 160-164.

OLINER JD, PIETENPOL JA, THIAGALINGAM S, GYURIS J, KINZLER KW AND VOGELSTEIN B. (1993). Oncoprotein MDM2 conceals the active domain of tumour suppressor TP53. *Nature*, **362**, 857-860.

PODCZASKI E AND HERBST AJ. (1986). Cancer of the vagina and the fallopian tube. In *Gynecologic Oncology*, Knapp RC and Berkowitz RS. (eds) pp. 399-424. Macmillan: New York.

PRIDE GL, SCHULTZ AE, CHUPREVISH TW AND BUCHLET DA. (1979). Primary invasive squamous carcinoma of the vagina. *Obstet. Gynecol.*, **53**, 218-225.

PURDIE CA, O'GRADY J, PIRIS J, WYLLIE AH AND BIRD CC. (1991). TP53 expression in colorectal tumors. *Am. J. Pathol.*, **138**, 807-813.

SHEFFNER M, MÜNGER K, BYRNE JC AND HOWLEY PM. (1991). The state of the p53 and retinoblastoma genes in human cervical carcinoma cell lines. *Proc. Natl Acad. Sci. USA*, **88**, 5523-5527.

SETO E, USHEVA A, ZAMBETTI GP, MOMAND J, HORIKOSHI N, WEINMANN R, LEVINE AJ AND SHENK T. (1992). Wild-type TP53 binds to the TATA-binding protein and represses transcription. *Proc. Natl Acad. Sci. USA*, **89**, 12028-12032.

TARONE RE AND WARE J. (1977). On distribution-free tests for equality of survival distributions. *Biomedica*, **64**, 156-160.

YU CC-W, WILKINSON N, BRITO MJ, BUCKLEY CH, FOX H AND LEVISON DA. (1993). Patterns of immunohistochemical staining for proliferating cell nuclear antigen and TP53 in benign and neoplastic human endometrium. *Histopathology*, **23**, 367-371.